# *Cracd* Marks the First Wave of Meiosis during Spermatogenesis and Is Mis-Expressed in Azoospermia Mice

**DOI:** 10.3390/jdb8030021

**Published:** 2020-09-18

**Authors:** Paige L. Snider, Olga Simmons, Simon J. Conway

**Affiliations:** Herman B. Wells Center for Pediatric Research, Indiana University School of Medicine, Indianapolis, IN 46033, USA; psnider@iupui.edu (P.L.S.); osimmons@iupui.edu (O.S.)

**Keywords:** *Cracd*, mouse testis development, spermatogonia, differentiation, gene expression, meiosis, β-catenin, azoospermia

## Abstract

Testicular development starts in utero and maturation continues postnatally, requiring a cascade of gene activation and differentiation into different cell types, with each cell type having its own specific function. As we had previously reported that the *Capping protein inhibiting regulator of actin (Cracd)* gene was expressed in the adult mouse testis, herein we examine when and where the β-catenin associated *Cracd* is initially expressed during postnatal testis development. Significantly, *Cracd* mRNA is present in both the immature postnatal and adult testis in round spermatid cells, with highest level of expression occurring during the first wave of meiosis and spermatogenesis. In the juvenile testes, *Cracd* is initially expressed within the innermost region but as maturation occurs, *Cracd* mRNA switches to a more peripheral location. Thereafter, *Cracd* is downregulated to maintenance levels in the haploid male germ cell lineage. As *Cracd* mRNA was expressed within developing round spermatids, we tested its effectiveness as a biomarker of non-obstructive azoospermia using transgenic knockout mice models. Meaningfully, *Cracd* expression was absent in *Deleted in azoospermia like (Dazl)* null testis, which exhibit a dramatic germ cell loss. Moreover, *Cracd* was abnormally regulated and ectopically mis-expressed in *Polypyrimidine tract binding protein-2 (Ptbp2)* conditional germ cell restricted knockout testis, which exhibit a block during spermatid differentiation and a reduction in the number of late stage spermatocytes coincident with reduced β-catenin expression. Combined, these data suggest that *Cracd* is a useful first wave of spermatogenesis biomarker of azoospermia phenotypes, even prior to an overt phenotype being evident.

## 1. Introduction

Spermatogenesis is a complex synchronized process where spermatogonia develop into highly differentiated spermatozoa through several tightly regulated transcriptional cascades and signaling pathways, involving many different types of cells, both somatic and germline. This unidirectional differentiation process starts with mitotic division of spermatogonial stem cells located close to the basement membrane of the tubules, giving rise to Type A spermatogonia that replenish the stem cells, and Type B spermatogonia that then divide to form early spermatocytes which undergo meiosis. After the long-lasting prophase of the first meiotic division, two secondary spermatocytes are produced, which, subsequently, meiotically divide into two equal haploid spermatids. In the last spermiogenesis differentiation phase, round spermatids undergo dramatic morphological and shape transformation to give rise to mature spermatozoa that are released into the seminiferous tubule lumen [1,2,3,4]. Throughout spermatogenesis and postnatal testicular remodeling, germ cells have extensive contact and communication with Sertoli cells (somatic cells that physically span the seminiferous epithelium) as well somatic cells outside the tubule, such as Leydig and peritubular-myoid cells [5]. The end goal is to ensure that germ cells within the seminiferous epithelium generate a continuous supply of haploid spermatozoa, which can then undergo spermiogenesis to form mature spermatozoa (sperm cells), thereby enabling efficient reproduction and preservation of the number of chromosomes in offspring [1,2]. However, a variety of conditions, classified as either ‘congenital’ and present at birth or ‘acquired’ as develops postnatally, can cause spermatogenic failure and infertility. Spermatogenic failure can result from hypothalamic, pituitary, or testicular disorders [6] and primary testicular disorders such as Oligospermia (abnormally low amount of sperm in semen) or Azoospermia (lack of measurable sperm in the semen) are responsible for causing the majority of male infertility.

Azoospermia, a condition in which there are no spermatozoa in the ejaculate, occurs in approximately 1% of all men and ranges between 10% and 15% among infertile men [7]. Moreover, azoospermia may occur because of an obstruction in the reproductive tract (called obstructive azoospermia) or the more prevalent condition of inadequate spermatozoa nor late-stage spermatids production (called non-obstructive azoospermia) which results in male infertility [8]. Given that non-obstructive azoospermia is most likely related to a failure in the spermatogenic process, we need to focus on identifying and further understanding the genes involved in testicular spermatogenesis and aberrant signaling pathways that cause male infertility. As the mammalian testis ranks among the top tissues with respect to transcriptome complexity, expressing the most (~84%) genes [5] and there are thought to be multiple mechanisms that may underlie non-obstructive azoospermia, detailed information regarding the cell-type-specific expression patterns of spermatogenic genes is needed to prioritize potential pathogenic variants that contribute to the pathogenesis of azoospermia [8]. Moreover, establishing when and where uncharacterized spermatogenic genes are expressed is of crucial importance to understanding or predicting their physiological role and how they may interact to form the complex expression networks that underlie spermatogenesis and testis development. We previously reported that the *Capping protein inhibiting regulator of actin (Cracd)* gene is expressed in the adult mouse testis [9]. Herein, we examine when and where *Cracd* is expressed during postnatal testis development, in order to begin to determine its function. The synchronous pattern of cell divisions during the first wave of spermatogenesis in mice has been widely used to study unknown gene requirement and potential gene function [3,4,5]. As *Cracd* can act as a β-catenin suppressor in cancer and *Cracd* global knockout induces intestinal epithelial cell integrity loss and WNT signaling deregulation [10], and the important and key role played by WNT/β-catenin signaling in spermatogenesis [11,12,13,14,15] and azoospermia [16], we carried out detailed spatiotemporal expression profiling of *Cracd* in both the normal postnatal mouse testis and within two azoospermia mouse models.

## 2. Materials and Methods

Mice. Normal testes were harvested from wild type C57BL/6J mice at postnatal (P) day 5- to 4-month-old (n = 3 samples/stage). Fixed and genotyped mouse testis were also provided by Dr. D.D. Licatalosi at Case Western Reserve University and isolated from *Dazl^Tm1hgu^* allele knockout and wildtype littermate mice (n = 3 mutants with wildtypes) on a 50:50 CD1: C57BL/6N background as described [17]. Dr. Prof. D. Licatalosi at Case Western Reserve University also provided fixed and genotyped testis isolated from conditional knockout and wildtype littermate mice (n = 7 mutants with wildtypes) derived from intercrossing *Stra8^-iCre+/+^; Ptbp2^ΔE4/+^* males with *PtbpT^flox/+^* females on a C57BL/6N background as described [18]. Animal procedures and experimental conditions were refined to minimize harm to animals and performed with the approval of the Institutional Animal Care and Use Committee of Indiana University School of Medicine (protocol #11364 approved 6/24/2020).

Quantitative PCR analysis of Cracd expression levels. Total RNA was isolated using RNEasy (QIAGEN, Germantown, MD, USA) kit from P5, 10, 16, 21, 4 week and 4-month-old isolated C57BL/6J mice testis (n = 3 samples/stage) and a 4-month-old epididymis negative control. mRNA was reverse transcribed using SuperScript II Reverse Transcriptase and cDNAs amplified within the linear range using two separate pairs of *Cracd* primers as described [9,19]. *Cracd* (initially called *Crad, mKiaa1211, C530008M17Rik,* MGI:2444817) primers were designed to amplify nucleotides 4251–4435 and 4452–4586, and, as both generated similar results (amplified products were sequenced to verify identity), the figure only illustrates 4452–4586 primer data. qPCR was performed in technical triplicate for each sample, and qPCR reactions were carried out using SYBR GreenER (Roche, Basel, Switzerland). Loading control and normalization was via comparison of *Cracd* with the geometric mean of the two most stable reference genes expressed during the various stages of mouse testis development [20]. This normalization factor was determined using the comparative ΔC_t_ method and PCR primers against *Peptidylprolyl isomerase A* (MGI:97749) and *Beta-actin* (MGI:87904) as described [20]. The relative quantification of *Cracd* gene expression between developmental stages, hearts, and/or organs was calculated by the 2^−ΔΔCt^ approximation method. All data are presented as means ± SEM. A two-tailed Student *t*-test on averaged change in threshold cycle values of the mRNAs was used. Differences were considered to be statistically significant for those with *p* < 0.05. Statistical analysis was performed with Prism software version 5.02 (GraphPad).

Histology and detection of mRNA and protein expression sites. Mouse testis were fixed in 4% paraformaldehyde overnight, dehydrated, embedded in paraffin, and sectioned at 5 and 10 μm. Thin 5 μm sections were stained with hematoxylin and eosin for histological analysis. For in situ hybridization, both sense and anti-sense non-radioactive RNA probes were synthesized from the cloned 432 base pair *Cracd* cDNA [9] and labeled with digoxigenin using the DIG RNA Labeling kit (Roche, Basel, Switzerland). Specific signal was only observed when sections were hybridized with the anti-sense probe, and serial sections were examined for comparable spatiotemporal patterns of *Cracd* expression in at least three consecutive serial sections at each stage. High power images are indicative of expression throughout the testis. For immunohistochemistry, serial sections were first antigen retrieved via microwave in citrate pH6 buffer, then probed with a rabbit monoclonal [E247] against β-Catenin (1:500 dilution, Abcam, Cambridge, MA, USA) and detected using the ABC kit (Vector, Burlingame, CA, USA) with DAB and hydrogen peroxide as chromogens following manufacturer′s directions. Antibody diluent (Agilent DAKO, Santa Clara, CA, USA), without primary antibody, was used for negative controls.

## 3. Results

### 3.1. Cracd mRNA Expression Levels in Postnatal Mouse Testis

**Figure 1:**
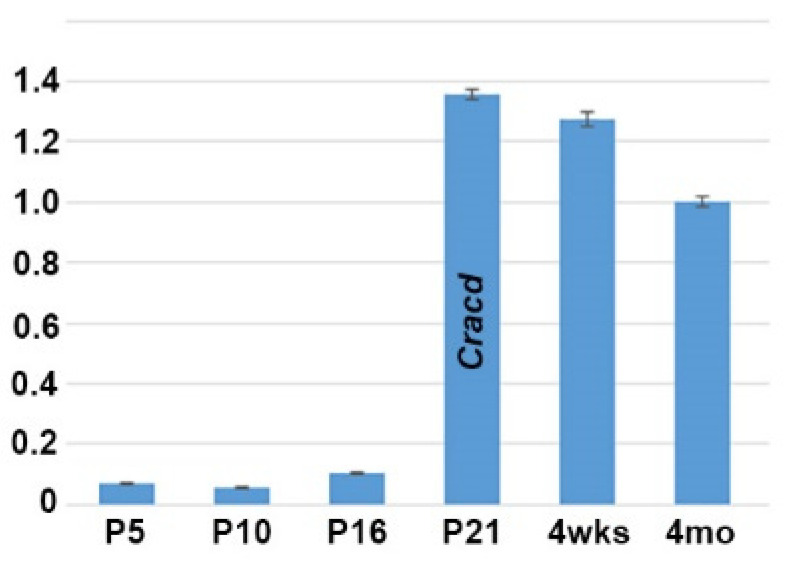
Quantitative PCR analysis of *Cracd* mRNA levels during postnatal testis maturation. Note *Cracd* levels are barely detectable in the P5 testis (10.1× fold relative to 4-month-old adult) and P10, with a slight increase in P16. However, at P21 *Cracd* levels are significantly upregulated (1.36× fold relative to 4-month-old adult) and stay elevated in 4-week-old and adult 4-month-old testis. Note, P21 *Cracd* levels are robust, as they are detectable at 23.3 cycles, compared to housekeeping reference genes *Ppia* and *Actb* at 16.87 and 19.03 cycles respectively (these are two most stable reference genes expressed during the various stages of mouse testis development [20]). Isolated 4-month-old epididymis was used as a negative control (not shown). qPCR data are presented as a logarithmic plot of relative expression, where a value of 1 indicates no difference in 4-month-old adult isolated and values <1 indicate reduced and >1 indicate increased expression. Error bars represent SD.

As we previously reported that *Cracd* (*Capping protein inhibiting regulator of actin*, MGI:2444817) is enriched and expressed in a defined pattern within the adult mouse testis, we used quantitative PCR (qPCR) analysis to determine when *Cracd* is initially expressed in the developing testis. We examined testes isolated from mice at P5, P10, P16, P21, 4 weeks and at adulthood because of the many known morphological and functional changes that occur in the juvenile testes as well as those at sexual maturity [1,4,21]. Significantly, *Cracd* mRNA is barely detectable at P5 (when spermatogonia first begin to differentiate) and P10 (when seminiferous tubules are mainly composed of Sertoli cells and spermatogonia), with a slight increase in *Cracd* is seen in P16 testis, when more than half of the seminiferous tubules already contain spermatocytes (Figure 1). However, at P21 (when the majority of the seminiferous tubules exhibit spermatocytes as the most advanced germ cell type but some round spermatids are present in some tubules) *Cracd* mRNA levels are significantly upregulated and present at a robust level (relative to P*pia* and *ActB* reference controls). Furthermore, robust *Cracd* levels were maintained in 4-week-old (when elongating spermatids appear) and in 4-month-old adult testis, albeit at slightly lower levels that P21 testis (Figure 1). Thus, *Cracd* mRNA is present in both the immature postnatal and adult testes, with highest level of expression occurring during the first wave of meiosis and spermatogenesis (between P21–P35) and in testis that have just begun the production of mature spermatozoa (which occurs between P30–35). Thereafter, *Cracd* mRNA continues to be expressed at relatively robust levels in the adult testis.

### 3.2. Cracd Spaciotemporal Expression in Mouse Testis

**Figure 2:**
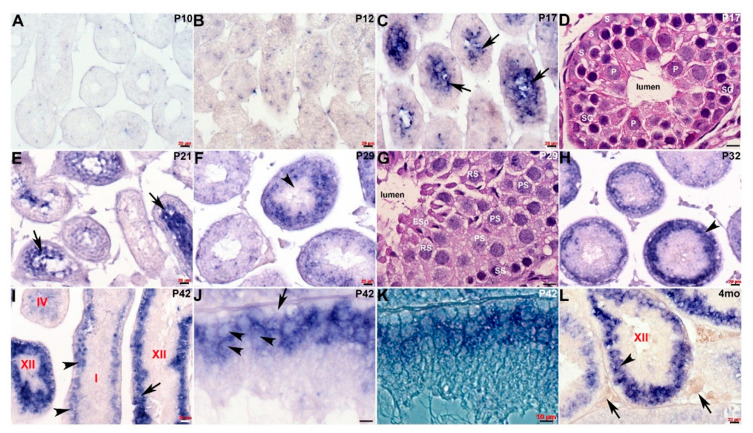
Spatiotemporal analysis of *Cracd* expression. (**A**–**C**) Non-radioactive in situ hybridization detection of *Cracd* mRNA (blue/purple precipitate) in staged postnatal mouse testis revealed very limited expression at P10 (**A**) and P12 (**B**), but a significant increase in levels within the innermost region in P17 seminiferous tubules (**C**, arrows). (**D**) Hematoxylin-eosin stained sections reveal that P17 seminiferous tubules contain mainly peripheral Sertoli cells (**S**) and spermatogonia, with spermatocytes in the pachytene stage (P) occupying most of the inner layer around the lumen. (**E**) In testis cross-sections showing a number of seminiferous tubules at different epithelial stages, *Cracd* continues to be expressed in inner regions at P21 (**E**, arrows). (**F**) However, thereafter there is a shift in site of *Cracd* expression to the periphery of the mature P29 seminiferous tubules, and Cracd is no longer expressed in inner cells (**F**, arrowhead). (**G**) Hematoxylin-eosin stained sections reveal that *Cracd* is expressed in P29 tubules in mainly peripheral primary (PS) and secondary spermatogonia (SS) and round spermatids and is absent from the inner elongating spermatids (ESp). (**H**–**L**) In P32 (**H**), P42 (**I**–**K**) and 4-month-old (**L**) testes, robust *Cracd* mRNA remains restricted to the sub-basal layer of germ cells (arrowheads, **H**,**I**,**L**) and is absent from the inner cells, spermatids and spermatozoa. At P42, in high power light field and phase images, *Cracd* is expressed around the large pachytene spermatocytes in Stage I and XII tubules (arrowheads, **I**,**J**,**L**) but is absent in basal layer of germ cells attached to the tubular wall basement membrane (arrow, **J**). Note interstitial (Leydig and blood/lymph vessels) cells (arrows, **L**) are negative for *Cracd*. Seminiferous tubule stages depicted in red. Scale bars **A**–**C**,**E**,**F**,**H**,**I**,**L** = 20 μm; **D**,**G**,**J**,**K** = 10 μm.

We previously demonstrated that *Cracd* mRNA is expressed in adult mouse testis within the germ cells [8]. To further define the transcriptional temporal profile and uncover when and where within mouse spermatogenesis *Cracd* mRNA is initially expressed, we used in situ hybridization to map *Cracd* in staged postnatal, juvenile (P5-4 weeks), and adult (4-month-old) C57BL/6J mice [1,21]. Both control sense and anti-sense digoxigenin-labeled cDNA probes were transcribed and used for non-radioactive in situ hybridization [9]. As expected, *Cracd* specific signal was only observed when sections were hybridized with the anti-sense *Cracd* probe and the same expression pattern was observed in three consecutive serial testis sections (Figure 2). *Cracd* expression was undetectable via in situ hybridization in mitotic P5 and P7 mouse testes, that contain only Sertoli cells (SCs) and mitotic spermatogonia in the seminiferous tubules. Note that qPCR detected a negligible *Cracd* signal at P5 (see Figure 1), but this is rather due to the heightened sensitivity of qPCR amplification rather than real *Cracd* mRNA expression. Significantly, during the initiation of meiosis at P10 (as the early spermatocytes appear and initiate leptotene stage of meiosis) the earliest *Cracd* mRNA in postnatal testis was detectable, as a few scattered individual cells expressed *Cracd*. A similar pattern was observed at P12 (zygotene stage of meiosis), but more cells express *Cracd* as the spermatocytes divide by meiotic division (Figure 2A,B). Significantly, *Cracd* expression levels are increased and are preferentially detected at the center of the P17 seminiferous tubules, in which mainly pachytene and diplotene stage spermatocytes are present [3]. Given that qPCR detected very little *Cracd* mRNA at P16, these P17 in situ data indicate *Cracd* is robustly upregulated within 24 h. Similarly, at initiation of spermiogenesis at P20-21, *Cracd-*positive spermatogenic cells are still preferentially found in the innermost layer, which contains mainly round spermatids undergoing meiosis (Figure 2C,E). This in situ data is in agreement with the qPCR data demonstrating that P21 testes exhibit robust levels of *Cracd* expression (see Figure 1). However, after the termination of meiosis, there is a change in localization of *Cracd* from the innermost region to a layer of germ cells (including primary, secondary spermatocytes, and round spermatids) at the P29 periphery (Figure 2F), as well as in the basal compartment in testis seminiferous tubules. This alteration in *Cracd* expression is more apparent at P32 (Figure 2H), as spermatids are completing the first wave of spermatogenesis and have already reached the elongation phase, and sperm tail accessory structures are being constructed [22]. The process of spermatogenesis is synchronized, with waves of differentiation periodically cycling through 12 epithelial stages (defined by the combination of germs cells present) along the length of each tubule [21]. *Cracd* expression exhibits a similar upsurge in mRNA coinciding with the highest expression levels in P42 Stage XII tubules (which are characterized by the presence of spermatocytes in either the first or second meiotic division and/or secondary spermatocytes), whilst in Stage I, *Cracd* is expressed in large pachytene spermatocytes but Stages V–VIII exhibit the lowest *Cracd* levels *(*Figure 2I–K). Significantly, using single cell RNA-sequencing in healthy fertile adult males (GEO: GSE120508), *CRACD* mRNA has also been reported to be localized to pachytene stage spermatocytes in the adult human testis transcriptional cell atlas [23]. In 4-month-old testis, *Cracd* expression is confined to a sub-basal layer of round spermatids and remains absent from the innermost elongating spermatids and spermatozoa, as well as peripheral spermatogonia, Sertoli cells, and basement membranes (Figure 2L). At this mature stage, roughly half of the spermatogenic cells are in the late spermatid stage. In contrast, *Cracd* is not expressed in adult interstitial (Leydig or peritubular-myoid) cells that synthesize and secrete male sex hormones, and blood and lymph capillaries are also negative for *Cracd* mRNA. Moreover, both the epididymis and ductus deferens that transfers the spermatozoa between epididymis and urethra are negative for *Cracd* expression (negative data not shown). Thus, during postnatal testis development and maturation, *Cracd* expression is confined to the first wave of spermatogenesis within the testis and germ cells but is absent in mature sperm, somatic and post-testicular sperm conducting tissues.

### 3.3. Cracd Expression Is Absent in DazL Knockout Testis

**Figure 3:**
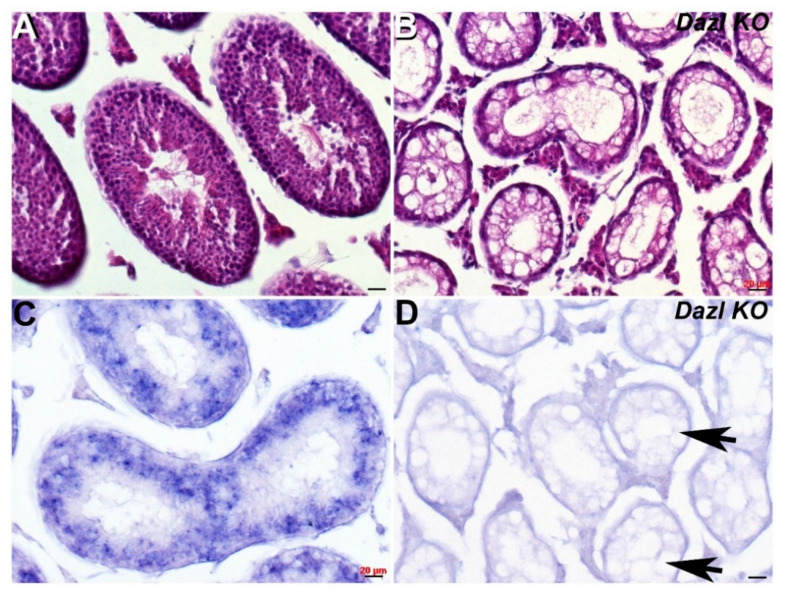
Spatiotemporal analysis of *Cracd* expression in *Dazl KO* testis. (**A**,**B**) Hematoxylin and eosin staining of P29 wildtype (**A**) and *Dazl* knockout (**B**) littermate testis, revealing numerous vacuoles (arrows, **D**) and the absence of any germ cells in *Dazl* null testis. Non-radioactive in situ hybridization detection of *Cracd* mRNA in P29 wildtype (**C**) and *Dazl* knockout (**D**) testis. Note, complete absence of *Cracd* in *Dazl* null testis but robust mRNA in wildtype control littermate. Scale bars = all 20 μm.

As *Cracd* mRNA expression is expressed within developing round spermatids (haploid male germ cells) during the first wave of spermatogenesis, we tested its effectiveness as a biomarker of nonobstructive azoospermia using transgenic knockout mice models. One of the most severe and well-studied models is the *Dazl* (*Deleted in azoospermia-like, MGI:1342328*) knockout, which leads to dramatic germ cell loss [24]. Dazl is thought to be required for initiation of translation and is expressed in the cytoplasm of pluripotent stem cells, and in both male and female germ cells, where it is essential for gametogenesis [24,25]. Using *Dazl* knockout mice in which most germ cells are lost by P6, we tested whether *Cracd* would be absent. Significantly, P29 *Dazl* null testes fail to exhibit any *Cracd* expression (Figure 3D), confirming that *Dazl* mutant nulls completely lack round spermatids and that *Cracd* may be a useful biomarker.

### 3.4. Cracd Expression Is delayed in Ptbp2 Conditional Knockout Tesis

As the *Dazl* knockout phenotype is so dramatic, we also tested whether *Cracd* mRNA would be informative in a less severe azoospermia model. Thus, we examined *Ptbp2* conditional knockout mutant testis, as conditional deletion of *Ptbp2* (*Polypyrimidine tract binding protein-2, MGI:1860489*) results in a block during spermatid differentiation and a reduction in the number of late stage spermatocytes [5]. As *Ptbp2* global nulls are perinatally lethal due to its requirement in binding intronic polypyrimidine clusters in pre-mRNA molecules [5,18], we used testes derived from intercrossed *Ptbp2* floxed and *Stra8-iCre* mice that resulted in *Cre/loxP* deletion of *Ptbp2* in spermatogonia from P3 onwards [26]. We examined both P21 and P42 *Ptbp2* cKO and wildtype littermate testis, to determine whether *Cracd* is mis-expressed prior to the known appearance of an overt abnormal phenotype and to test if *Ptbp2* cKO loss affects pachytene stage spermatocytes, round spermatids and/or *Cracd* expression.

**Figure 4:**
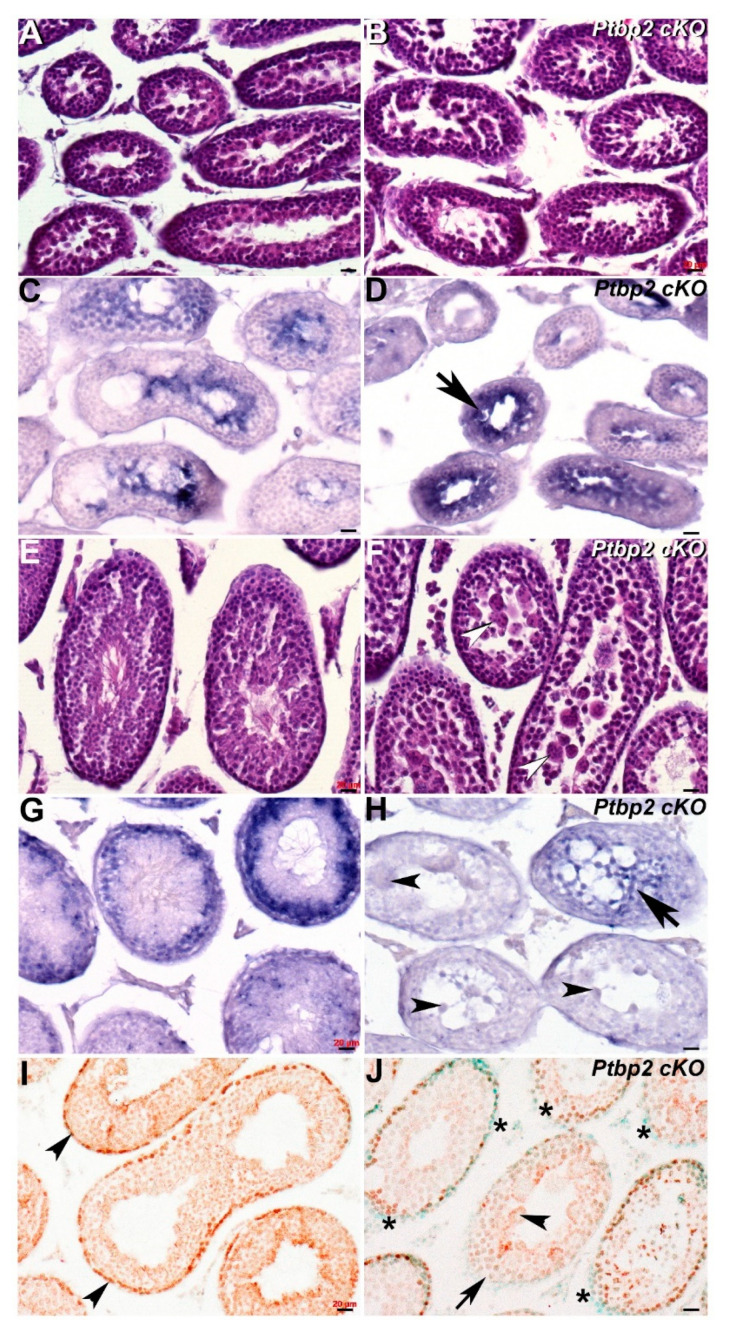
Spatiotemporal analysis of *Cracd* expression in *Ptbp2 cKO* testis. (**A**,**B**) Hematoxylin and eosin staining of P21 wildtype (**A**) and *Ptbp2* conditional knockout (**B**) littermate testis. Non-radioactive in situ hybridization detection of *Cracd* mRNA in P21 wildtype (**C**) and *Ptbp2* cKO (**D**) testis, revealing that Cracd expression is more confined and upregulated (arrow, **D**). (**E**,**F**) Hematoxylin and eosin staining of P42 wildtype (**E**) and *Ptbp2* conditional knockout (**F**) littermate testis. Note smaller tubule size and multiple giant multinucleated cells evident (arrowheads, **F**). Non-radioactive in situ hybridization detection of *Cracd* mRNA in P42 wildtype (**G**) and *Ptbp2* cKO (**H**) testis, revealing *Cracd* expression is significantly diminished (arrow, **H**) and *Cracd* is not expressed in multiple giant multinucleated cells (arrowheads, **H**). (**I**,**L**) Immunostaining using monoclonal β-catenin antibody (signal is brown DAB precipitate) counterstained with methyl green showed β-catenin expression in P42 wildtype (**I**) and *Ptbp2* cKO (**J**) testis sections. Note uniform nuclear localization in peripheral cells in wildtype (**I**, arrowheads) but most *Ptbp2* cKO seminiferous tubule peripheral cells exhibit reduced β-catenin expression (**J**,*). Moreover, the *Ptbp*2 cKO tubule with multiple giant multinucleated cells (arrowheads, **J**) is absent of β-catenin expression (arrowhead, **J**). Scale bars = all 20 μm.

Significantly, although histological examination revealed that P21 *Ptbp2* cKO and wildtype littermate testis are similar, *Cracd* mRNA expression is present in a more restricted pattern and is upregulated in P21 cKO testis (Figure 4B,D). This may suggest that there is an accumulation of abnormal pachytene stage spermatocytes and/or round spermatids in early *Ptbp2* cKO tubules. However, in contrast, when an overt phenotype is present and multiple giant multinucleated cells evident, *Cracd* mRNA expression is significantly reduced and/or absent (Figure 4H). Moreover, following the termination of meiosis, there is no switch in localization of *Cracd* from the innermost region to a layer of round spermatids to the periphery as normally seen in wildtype littermates, in P42 cKO testis (Figure 4F,H). In order to test whether the WNT/β-catenin-linked *Cracd* mRNA alterations are indeed indicative of altered β-catenin expression and or WNT/β-catenin signaling, we used immunohistochemistry to examine β-catenin localization. Significantly, when *Cracd* mRNA expression is present in peripheral region of the seminiferous tubules, there is robust nuclear localization of β-catenin in peripheral cells (usually proliferative at this stage of development [27]) in P42 wildtype testis, revealing active canonical WNT signaling (Figure 4I). However, when *Cracd* mRNA is absent or ectopically localized in the middle of the tubules, there is either reduced and/or absent peripheral β-catenin nuclear expression in P42 *Ptbp2* cKO testis (Figure 4J). Thus, *Cracd* may indeed be a useful first wave of spermatogenesis biomarker of subtle azoospermia phenotype, even prior to an overt phenotype being evident. Moreover, misexpression of *Cracd* mRNA marker via reduction and/or ectopic localization is indicative of altered β-catenin expression, and possibly WNT signaling itself. *Cracd* downregulation is also indicative of when haploid germ cells are lost due to the increased apoptosis [5,18], as observed in abnormal meiotic spermatocytes and post-meiotic arrest of abnormal *Ptbp2* cKO spermatid differentiation.

## 4. Discussion

The aim of this study was to clearly determine when and where *Cracd* mRNA is expressed in the developing postnatal testis and to test whether its spaciotemporal expression pattern informs us of its function and/or whether it will be useful in screening for spermatogenic failure. Undeniably, our expression analysis revealed that *Cracd* is specifically upregulated during the P17 first wave of spermiogenesis and that variance in *Cracd* spaciotemporal patterns and intensity within the round spermatids correlates with the synchronized processes of spermatogenesis (see Figure 1; Figure 2). Significantly, *Cracd* expression exhibits an upsurge in mRNA coinciding with the highest expression levels in Stage XII tubules and *Cracd* is robustly expressed in large pachytene spermatocytes. Moreover, in 4-month-old testis, *Cracd* mRNA is present at maintenance levels in the peripheral pachytene stage spermatocytes and round spermatids but is switched off as the spermatids migrate toward the seminiferous tubule lumen and reach the elongation phase and when sperm tail accessory structures are being constructed. However, *Cracd* mRNA is not expressed in the somatic interstitial Leydid/peritubular-myoid cells, blood/lymph vessels, nor epididymis or ductus deferens. Combined, this clearly demonstrates that *Cracd* is a specific mouse testicular germ cell biomarker. Moreover, *CRACD* mRNA has also been reported to be localized to pachytene stage spermatocytes in the adult human testis transcriptional cell atlas [23]. However, we fully appreciate that these are mRNA data and that it will be important to also examine protein localization (when a commercial antibody becomes available), as mRNA and protein expression do not always overlap. Similarly, as we cannot exclude the possibility that very low level *Cracd* mRNA is present in the early neonatal testis or within a few somatic cells, a specific antibody would help with these remaining questions. Nevertheless, due to the synchronized progression of the first wave of spermatogenesis, the postnatal testis provides an excellent model system to study *Cracd* gene expression during germ cell development.

Next, we sought to test whether *Cracd* was a useful biomarker and therefore examined its expression patterns in two diverse mouse models of azoospermia. The finding that *Cracd* mRNA is absent in *Dazl* knockout was expected, given the known dramatic phenotype wherein there is a complete neonatal absence of germ cells in *Dazl* null testis [17,24,25]. These data confirm that *Cracd* is a good marker of round spermatids/germ cell lineage and that despite the *Dazl* null testis surviving past the 4-week-stage, there is no persistence nor reemergence of any *Cracd*-positive cells (see Figure 3). However, in this context, *Cracd* may be useful for screening of differentiated round and pachytene cells in the ejaculate or for confirmation of the presence of germ cells in biopsies. Thus, our finding that *Cracd* was both over-expressed and then subsequently under-expressed in the less drastically affected *Ptbp2* cKO testis of azoospermia mouse model, confirms its usefulness. Significantly, *Ptbp2* cKO and wildtype littermate control testes are comparable in size and weight until ~P33, when cKO testes thereafter show a marked reduction in the average testis weight/body weight ratio and an increase in seminiferous tubule cell apoptosis [5]. Moreover, prior analysis revealed that P21 and P25 cKO testis exhibit defective alternative splicing of overlapping mRNA targets prior to a phenotype being evident, suggesting that Ptbp2 is required P21 onwards. Furthermore, the finding that *Ptbp2* conditional knockout testis upregulate *Cracd* prior (P21) to the appearance of an overt structural phenotype (see Figure 4), suggests that germ cell-restricted deletion of the polypyrimidine tract binding Ptbp2 protein may initially either fail to post-transcriptionally regulate the entire spermatogenic transcriptome (including *Cracd*) resulting in prolongation of the pachytene stage of meiosis, that Ptbp2 may itself regulate *Cracd* intronic RNA regulatory elements or downstream control sequence or that *Ptbp2* mutant testis have an initial WNT/β-catenin signaling defect. Indeed, a Ptbp2/Sfpq (Ptb-associated splicing factor) complex has been shown to be involved in cancer metastasis involving activation of β-catenin signaling [28]. Significantly, one of the most prominent later structural features in P42 *Ptbp2* cKO tubules is the presence of round spermatids in the lumen, most commonly as giant multinucleated cells containing several spermatid nuclei [5]. Thus, our finding that *Cracd* expression levels are both suppressed and ectopically located in P42 mutant *Ptbp2* testis, suggests that ultimately the loss of *Ptbp2* results in a block in spermatogenesis that prevents round spermatids from undergoing spermiogenesis. Whereas, the P42 reduced *Cracd* levels are most likely a reflection of germ cell apoptosis known to occur in *Ptbp2* conditional knockout testis [5], the correlation that nuclear β-catenin localization is diminished and/or absent in *Ptbp2* cKO tubule periphery, suggests the novel idea that ubiquitous Ptbp2 may be required for normal WNT/β-catenin signaling during spermatogenesis. Determining which of these possibilities occurs and whether WNT/β-catenin signaling is affected will be the subject of our future studies, as this *Ptbp2* conditional knockout mouse model is a powerful tool to investigate the biologic and molecular roles of *Cracd* in an in vivo model system of mammalian azoospermia. Moreover, these gene expression studies have demonstrated the usefulness of *Cracd* biomarker as a tool to investigate the underlying WNT/β-catenin signaling-dependent azoospermia mechanisms.

As WNT/β-catenin signaling is a highly conserved cell-to-cell communication mechanism [12,13,14,15] and *Cracd* can function as a β-catenin suppressor [10], *Cracd* misexpression could be indicative of altered β-catenin expression (see Figure 4J). Thus, is tempting to speculate as to whether *Cracd* may play a role in WNT/β-catenin signaling within the postnatal testis. Although it is known that β-catenin is highly expressed in fetal Sertoli and germ cells of mice [14], that several Wnts are expressed in highly specific expression patterns in the seminiferous tubules [15], that spermatogonial stem cells require various Wnt ligands [29,30,31] and express Axin2 (as a marker of WNT/β-catenin pathway-responsive cells) in mice testis [15]; there is still limited information regarding the importance of WNT signaling in the postnatal testis. Despite this, these informative studies have led to the suggestion that the β-catenin complex binding Sertoli and germ cells to each other, may trigger a signaling cascade that regulates post-meiotic germ cell differentiation [14] and that undifferentiated spermatogonia initially require Wnt6 ligand during morphogenesis [15]. Indeed, β-catenin translocates to the nucleus when it is stabilized (i.e., low level of phosphorylation) and is most often involved in the regulation of cell adhesion and coupling of cadherins to the actin cytoskeleton. As Cracd is a capping protein inhibiting regulator of actin dynamics and may positively regulate actin polymerization [10], it is noteworthy that important actin-binding proteins (*Actin capping protein (CP) α3* and *β*3) are known biomarkers of male infertility in patients [32]. Similar to *Cracd*, both testis-specific CPα3 and CPβ3 are robustly expressed in human spermatocytes at the pachytene stage and are diminished in infertile spermatids and sperms [33]. Indeed, cytoskeletal elements are essential for morphological shape reorganization and movement of spermatogenic cells from the base of the seminiferous tubule toward the luminal edge during spermatogenesis, with specifically the actin filaments being concentrated in specific regions of spermatogenic cells and Sertoli cells [34]. Actin is involved in the crucial phases of spermatogenesis, and the altered expression of testis-specific actin capping proteins is suggested to be a cause of male infertility in humans [32]. Cracd itself may play a putative role in WNT/β-catenin driven actin regulation in germ cells during spermatogenesis and postnatal testicular remodeling. Thus, its misexpression in azoospermia models could be a useful indicator of disruption of the cadherin–catenin–actin complex. Unfortunately, although there are two *Cracd* mouse mutants, one is a hypomorph that exhibits normal testis and fertility [9] and the other is a *CRISPR/Cas9* mutant [10] in which the phenotype and/or fertility of homozygous males is unfortunately unknown. As it remains unclear if there are any observable structural abnormalities and whether a complete null *Cracd* allele would result in an abnormal phenotype, it is difficult to draw definitive conclusions regarding the genetic requirement of *Cracd* in the postnatal testis.

Up to 2% of the world′s men lack measurable sperm in their semen, with azoospermia representing the most frequent cause of male infertility. Elucidation of the molecular patterns and cellular locations of biomarker spermatogenesis genes is an essential first step in understanding the underlying mechanisms causing azoospermia and the eventual development of therapeutic options for this group of patients. As it has been estimated that more than 2000 genes may be involved in the maintenance of the germ cell population and appropriate accomplishment of meiosis [35], future protein, transgenic reporter, gain- and loss-of-function transgenic and functional requirement studies will be needed to determine the specific role of Cracd in spermatogenesis and if/how it may affect aspects of WNT/β-catenin signaling. These spatiotemporal expression analyses will help to further the understanding of this useful biomarker in normal postnatal testis development and to pave the way for examination of infertility defect pathogenesis.
